# Unexpected vasogenic oedema and alexia as complications after dural arteriovenous fistula embolization

**DOI:** 10.1093/bjrcr/uaaf012

**Published:** 2025-03-12

**Authors:** Yun-Hsien Ho, Hsin-Fan Chiang, Cheng-Chih Hsieh, Shih-Yang Wei, Chun-Chao Huang

**Affiliations:** Department of Medicine, Mackay Medical College, New Taipei City 252, Taiwan; Department of Medicine, Mackay Medical College, New Taipei City 252, Taiwan; Department of Radiology, Mackay Memorial Hospital, Taipei 104, Taiwan; Department of Medicine, Mackay Medical College, New Taipei City 252, Taiwan; Department of Radiology, Mackay Memorial Hospital, Taipei 104, Taiwan; Department of Medicine, Mackay Medical College, New Taipei City 252, Taiwan; Department of Radiology, Mackay Memorial Hospital, Taipei 104, Taiwan; Department of Medicine, Mackay Medical College, New Taipei City 252, Taiwan; Department of Radiology, Mackay Memorial Hospital, Taipei 104, Taiwan

**Keywords:** dural arteriovenous fistula, embolization, venous infarction, alexia

## Abstract

A 63-year-old male presented with acute onset of intermittent dizziness, visual disturbances, and left temporal headache. Investigations revealed a dural arteriovenous fistula (dAVF) at the left sigmoid sinus, classified as Cognard type IIb. Successful therapeutic transvenous embolization was performed using coils and Onyx, resulting in complete resolution of the dAVF without immediate complications. However, 3 days post-embolization, the patient developed headache, dizziness, visual discomfort, and alexia. MRI findings suggested vasogenic oedema in the left temporo-occipital area due to venous outflow obstruction. Despite treatment with enoxaparin and dexamethasone, the patient experienced progressive symptoms including difficulty in object naming, memory decline, and nonconvulsive seizures. Follow-up imaging indicated improvement of oedema and stable minimal focal gliosis. This rare case of a patient developing alexia following endovascular embolization of a dural AVF highlights the importance of post-procedural monitoring and suggests potential benefits of prophylactic anticoagulation to reduce the risk of probable complications.

## Clinical presentation

A 63-year-old male presented with acute-onset intermittent dizziness and hemi-ring visual disturbances under flashlight illumination. He developed a persistent left temporal headache lasting approximately 40 min over several days, aggravated by exercise but relieved with acetaminophen. There were no changes in colour saturation, light intensity, or postural correlation. The patient denied photophobia, phonophobia, neurological deficits, or a family history of headaches. Additional symptoms included pulsatile low-pitch tinnitus, bilateral muscle cramps, and nasal stiffness over the past 2 weeks.

## Investigation/image findings

Physical examination revealed left post-auricular bruits. Doppler ultrasound showed low-resistance flow in the left external carotid artery (ECA). Electroencephalography (EEG) detected rhythmic spiky discharges over the left hemisphere, peaking at T3T5, suggesting a non-convulsive seizure. Anti-epileptic therapy was thereby initiated.

**Figure 1.**
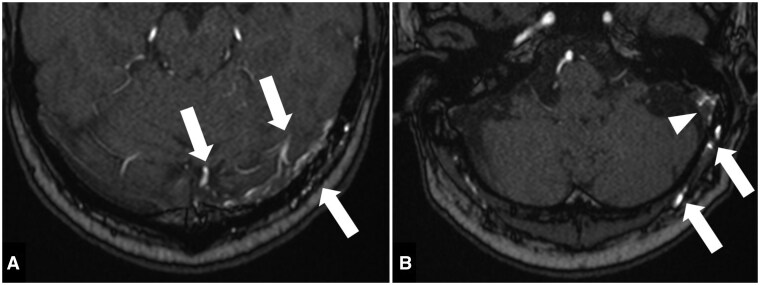
Time-of-fight magnetic resonance angiography (TOF MRA) showed tortuous vessels at the left parieto-occipital region and left occipital scalp (arrows in A and B) and abnormal signals in the left sigmoid sinus (arrowhead in B).

**Figure 2.**
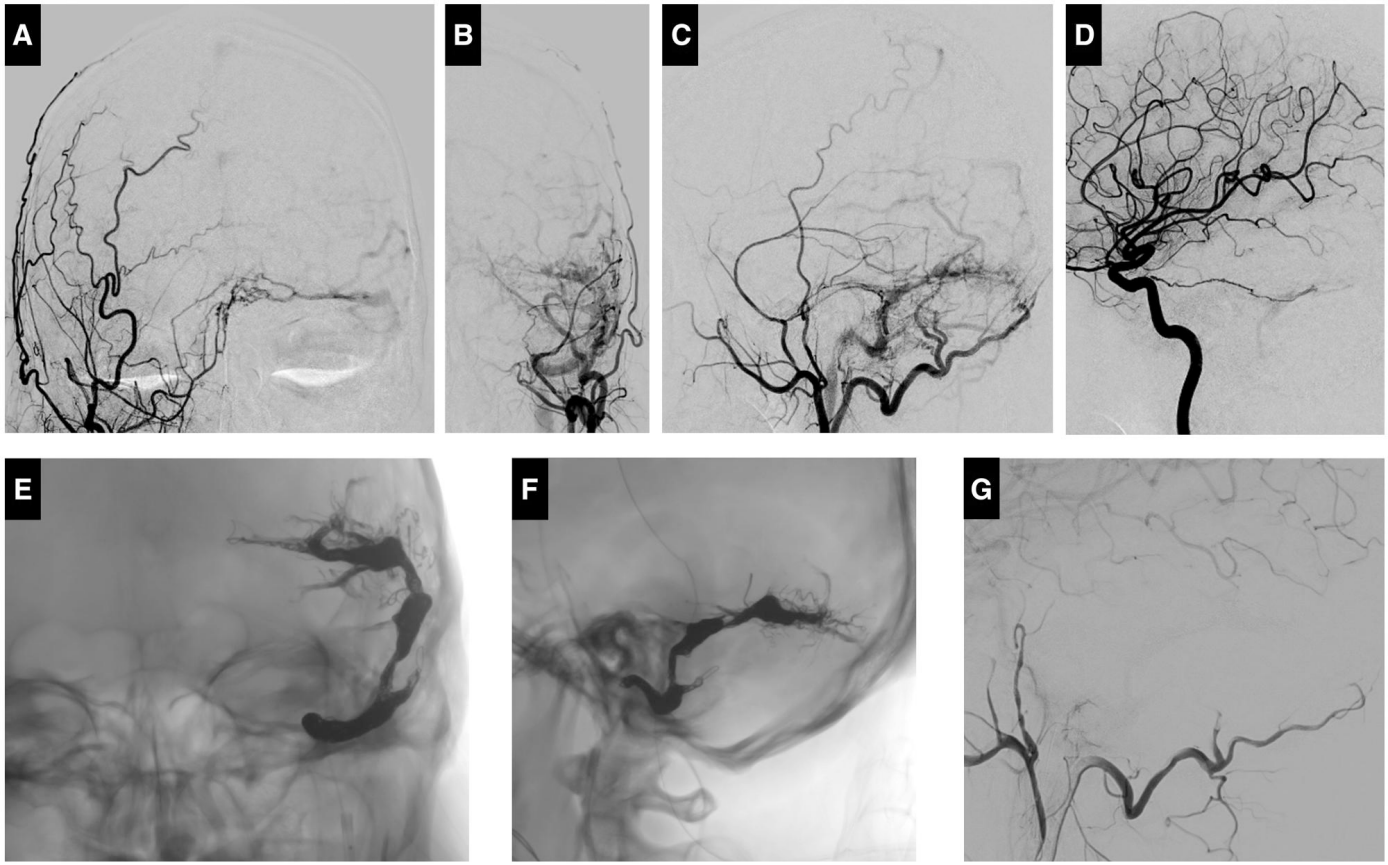
Cerebral angiography and transvenous embolization. Diagnostic cerebral angiography from (A) right external carotid arteriography on the antero-posterior view, (B and C) left external carotid arteriography on the antero-posterior view and lateral view, and (D) left internal carotid arteriography on the lateral view demonstrated abnormal arteriovenous shunting from bilateral occipital arteries, left middle meningeal artery, and left meningohypophyseal trunk to the left sigmoid sinus. (E and F) Transvenous embolization was conducted by using coils and Onyx 34 with evidence of Onyx penetration of feeding arteries and cortical veins. (G) Left external carotid arteriography on the lateral view after embolization revealed total occlusion of the arteriovenous fistula.

MRI unveiled tortuous vessels at the left parieto-occipital region and left occipital scalp, along with abnormal signals in the left sigmoid sinus (Figure 1) and suspected arteriovenous shunting. Diagnostic cerebral angiography confirmed a dural arteriovenous fistula (dAVF) at the left sigmoid sinus (Figure 2A-D), with arterial feeders originating from branches of both ECAs and the left meningohypophyseal trunk. Multiple shunting points were noted at the medial left transverse sinus, left transverse-sigmoid sinus junction, and the mid-sigmoid sinus, with venous drainage into multiple cortical veins and internal jugular vein, classifying the condition as Cognard type IIb.

## Treatment

To prevent haemorrhage, transvenous embolization was performed successfully using coils and 3 vials of Onyx 34 (Figure 2E-F). Post-procedural angiography disclosed complete resolution of the arteriovenous fistula (Figure 2G). On-table CT showed no haemorrhage. The patient remained stable, without headaches, nausea, vomiting, or signs of increased intracranial pressure.

## Complications and long-term outcome

**Figure 3.**
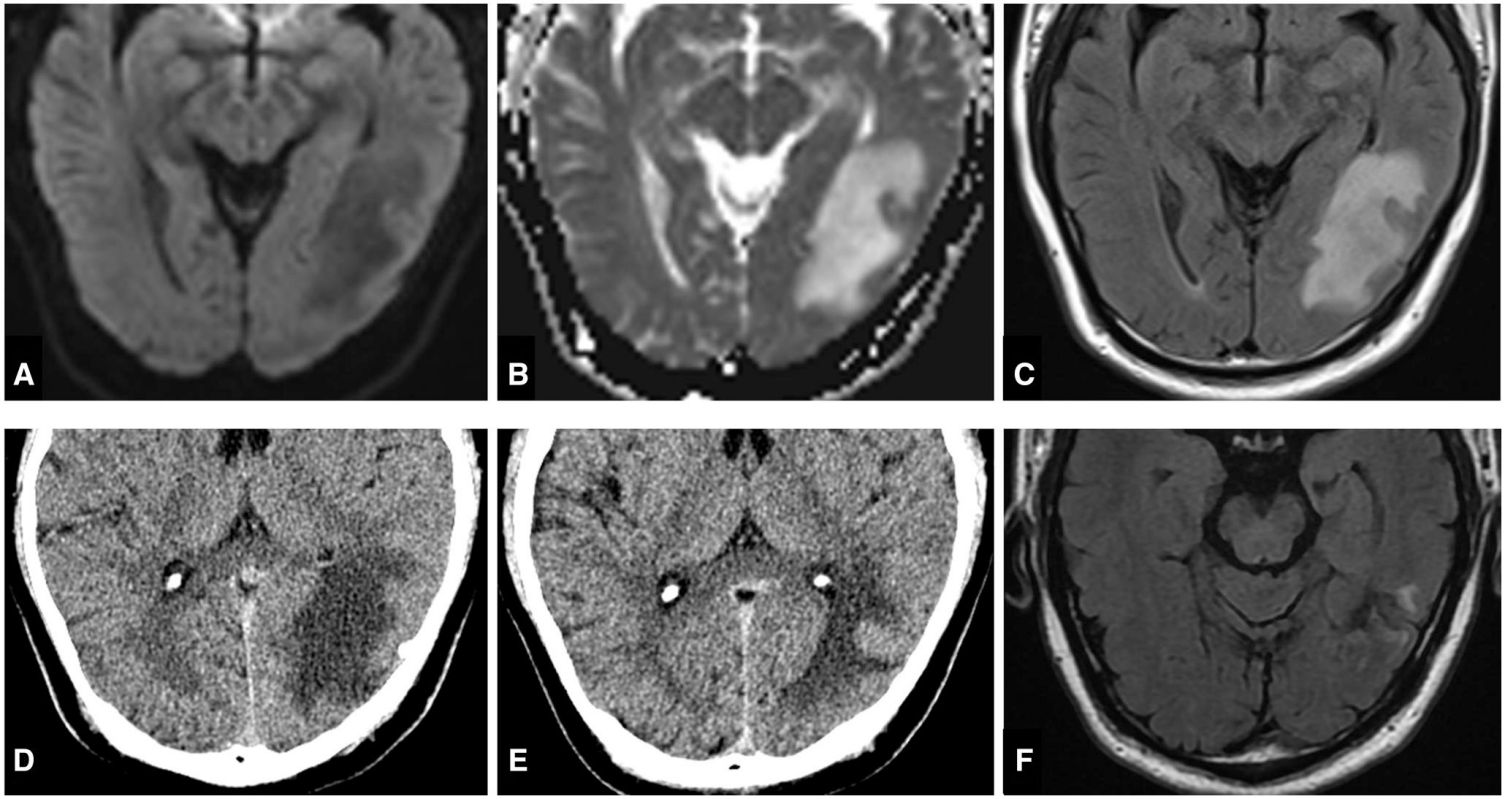
(A) Diffusion weighted imaging (DWI), (B) Apparent diffusion coefficient (ADC), and (C) T2-fluid-attenuated inversion recovery (T2-FLAIR) images 3 days after embolization revealed regional low DWI signal and high ADC value and T2FLAIR signal at left temporooccipital white matter. (D) CT images displayed white matter low densities at the same region. After treatment with enoxaparin and dexamethasone, (E) follow-up CT images in 2 weeks demonstrates gradually decreasing white matter oedema. (F) Follow-up T2-FLAIR images in 3 months showed minimal residual gliosis.

Three days later, the patient developed headaches, mild dizziness, visual discomfort, and alexia, with impaired reading and writing but preserved comprehension and speech. MRI demonstrated T2-FLAIR hyperintensity without diffusion weighted imaging (DWI) hyperintensity over the left temporo-occipital area (Figure 3A-C), suggesting vasogenic oedema due to venous outflow obstruction. Despite enoxaparin and hydration, symptoms progressed to naming difficulties, memory decline, reduced speech fluency, and non-convulsive seizures.

CT revealed post-embolization white matter changes in the left parieto-occipital lobes and focal hypodensity in the left capsular region (Figure 3D). Dexamethasone improved symptoms, enable discharge. Follow-up CT at 2 weeks showed mild resolution (Figure 3E).

At 1 month, reading and speech fluency returned to baseline, with gradual improvement in writing over 3 months. Muscle cramps resolved with reinstated anti-epileptic medication. MRI after 3 months of treatment showed resolution of oedema and minimal gliosis (Figure 3F), remaining stable after 1 year.

## Discussion

Dural arteriovenous fistulas, which only account for 0.1% of the population, are direct connections between arterial branches and dural venous sinuses. High venous pressure bypassing capillaries can lead to aggressive symptoms such as intracranial haemorrhage (40%-60%), seizure (10%-30%), headache (1%-6%), and other focal neurological deficits (1%-3%). High-grade dAVFs with cortical venous drainage warrant active interventions, primarily through endovascular embolization, with microsurgery reserved for refractory cases and radiosurgery for low-grade lesions. However, embolization carries risks, including ischaemic stroke, cranial nerve palsy, venous bleeding, and infarction.^1^

Onyx is widely used in embolization due to its deep penetration, but excessive venous occlusion could occasionally occlude functional cerebral veins, leading to potential venous infarction. Infarction may also result from extensive transvenous sinus packing, which completely blocks the sinus and obstructs normal antegrade cerebral venous outflow.^2^

Venous thrombosis is a known complication of endovascular embolization. A study of 115 intracranial dAVF patients treated with transarterial embolization found that 1 patient (0.9%) experienced permanent morbidity from left extremity weakness due to venous thrombosis, while 4 patients (3.5%) had transient neurological worsening.^3^ However, cases of post-embolization alexia is rarely reported. The first reported case in 2024 described a 60-year-old male with a Borden type III dAVF who developed transient word-finding difficulty after embolization. MRI revealed a left temporal subcortical infarction, likely caused by impaired venous drainage.^4^

In our patient, venous occlusion likely caused infarction in the left parietal and temporal lobes, resulting in vasogenic oedema. Unlike arterial infarcts, venous infarction presents with cortical sparing and non-arterial distribution involvement. Symptoms of venous infarction can be varied, including headache, blurred vision, and seizures. Moreover, dyslexia in this case suggests central nervous system impairment in the left temporal lobe, while memory loss suggests involvement of the hippocampus and parahippocampus.^5^

**Figure 4.**
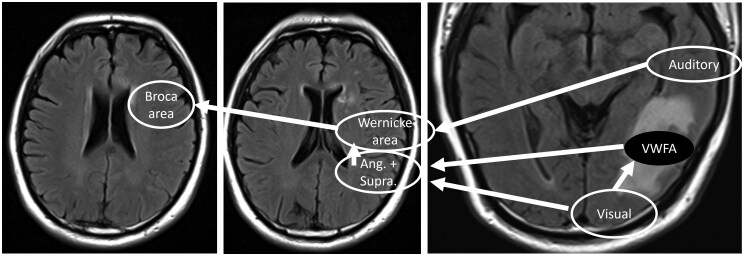
Diagram of alexia in this patient. Reading ability begins with visual input reaching the primary visual cortex (Visual). From there, the information is processed by the visual word form area (VWFA) and the angular and supramarginal gyri (Ang. + Supra.) for word recognition. Next, Wernicke’s area facilitates comprehension, while Broca’s area is responsible for speech production. Due to oedema, the VWFA is impaired, leading to the patient’s difficulty in reading. However, the auditory centre (Auditory) remains unaffected, allowing the patient to understand spoken language.

The process of reading begins with visual input reaching the primary visual cortex in the occipital lobes. From there, the information is relayed to the angular gyrus and supramarginal gyrus in the left inferior parietal lobe, as well as the visual word form area (VWFA) in the left ventral temporal lobe, which is crucial for word recognition. Next, Wernicke’s area in the posterior superior left temporal lobe facilitates comprehension, while Broca’s area in the left frontal lobe is responsible for speech production. In this case, the patient’s dyslexia-like symptoms are likely due to impairment of the VWFA, which has been affected by the oedema. However, since the auditory centre in the superior temporal lobe remains intact, the patient is still able to understand spoken language (Figure 4). Writing ability follows a complex neural pathway and may be associated with alexia. Specifically, alexia with agraphia has been linked to VWFA lesions. Therefore, the oedema affecting the VWFA may account for the patient’s overall symptoms.

Cerebral venous thrombosis is typically managed with anticoagulation, starting with heparin or low-molecular-weight heparin (LMWH) and transitioning to oral vitamin K antagonists for 3-12 months.^6^ While prophylactic anticoagulation after dAVF embolization remains debated, several studies support its necessity. For instance, a case study presented 2 patients with AVF treated successfully with surgical and endovascular embolization. Both patients developed massive cerebral venous thrombosis on brain imaging after several days; 1 patient died, and the other was severely disabled.^7^ Another retrospective study of 19 AVF patients treated with coil embolization reported postinterventional ophthalmic venous thrombosis in 2 patients, for which adequate anticoagulation is recommended.^8^ Similarly, in a case of non-galenic pial arteriovenous fistula in the posterior fossa that underwent complete embolization, steroids and LMWH were prescribed for 1 week post-embolization to prevent thrombosis in the draining veins.^9^

## Conclusion

This case illustrates a rare presentation of alexia following dAVF embolization due to venous infarction. Despite successful embolization, the following complications highlight the need for post-procedural monitoring and management. Prophylactic anticoagulation may reduce the risk of thrombosis and its sequelae. Further research on prophylactic anticoagulation treatment is needed to improve patient outcomes.

## Learning points

- Acute onset intermittent dizziness, visual disturbances, and specific headache may indicate underlying vascular abnormalities such as dural arteriovenous fistulas (dAVF).
- Patients with high-grade AVF should receive active interventions, including endovascular embolization, microsurgery, and radiosurgery.
- Regular follow-up and monitoring through imaging and clinical assessments are important for detecting delayed post-embolization complications.
- Clinicians should be aware of potential complications following endovascular embolization, such as vasogenic oedema and alexia.
- Anticoagulation must be used when post-procedural venous thrombosis develops, while adding corticosteroids may improve patient outcomes when symptoms progress.
- There may be potential benefits of using prophylactic anticoagulation to reduce the risk of venous thrombosis and related complications after dAVF embolization.
